# Ibrutinib and acalabrutinib use and risk of incident atrial fibrillation: a propensity-matched analysis

**DOI:** 10.1186/s40164-025-00619-6

**Published:** 2025-03-04

**Authors:** Joachim Alexandre, Jonaz Font, Thibault Lenormand, Sylvain Chantepie, Hippolyte Bardet, Gandhi Damaj, Charles Dolladille, Damien Legallois, Angélique Da-Silva, Paul Milliez, Arnaud Bisson, Laurent Fauchier

**Affiliations:** 1 Normandie Univ, UNICAEN, INSERM U1086 ANTICIPE, Biology-Research Building, Avenue de La Côte de Nacre, 14000 CAEN, France; 2 Department of Pharmacology, Biology-Research Building, Caen-Normandy University Hospital, PICARO Cardio-Oncology Program, Avenue de La Côte de Nacre, 14000 CAEN, France; 3 Department of Cardiology, Caen-Normandy University Hospital, Avenue de La Côte de Nacre, 14000 CAEN, France; 4 Service de Cardiologie, Centre Hospitalier Universitaire Trousseau, Tours, France; 5 Caen-Normandy University Hospital, Hematology Institute, Avenue de La Côte de Nacre, 14000 CAEN, France; 6 Department of Cardiology, Caen-Normandy University Hospital, PICARO Cardio-Oncology Program, Avenue de La Côte de Nacre, 14000 CAEN, France; 7 Departments of Pharmacology and Medical Oncology, Caen-Normandy University Hospital, PICARO Cardio-Oncology Program, Avenue de La Côte de Nacre, 14000 CAEN, France; 8 Normandie Univ, UNICAEN, INSERM U1237 PhIND, GIP Cyceron, Boulevard Henri Becquerel, 14000 CAEN, France

**Keywords:** Atrial fibrillation, Ibrutinib, Acalabrutinib, B-cell malignancies, Database

## Abstract

Ibrutinib and acalabrutinib are both associated with an increased risk of atrial fibrillation (AF); however, the comparative risk of AF between these 2 BTK inhibitors remains largely unknown. Our primary aim was to evaluate the risk of incident AF in patients exposed to ibrutinib compared to those exposed to acalabrutinib. Using the TriNetX research network database, we established a retrospective cohort of adult patients (≥ 18 years) previously diagnosed with a B-cell malignancy (using ICD-10-CM codes) in whom a first BTKi introduction occurred between January 1st, 2013 (first patient exposed to ibrutinib in TriNetX) and July 1st, 2024. Patients were divided into 2 groups based on their exposure to ibrutinib or acalabrutinib. After propensity score matching (PSM) across 37 covariates, Cox proportional hazard models were used to calculate the hazard ratios (HRs) and 95% confidence intervals (CIs) to compare the 2 matched groups. The appropriateness of the proportional hazard assumption was examined and risk differences (RDs) were used if appropriate. Results were summarized with the use of Kaplan–Meier survival curves. Follow-up started from 1 day after first BTKi introduction and continued over a 6-year follow-up period. A cohort of 12,449 patients exposed to ibrutinib and 4,131 to acalabrutinib were included in the study. After PSM, 4,090 patients remained in each group (1:1). During a mean duration of BTKi exposure of 2.3 ± 1.8 years, we found a significantly higher risk of incident AF in the ibrutinib group compared to the acalabrutinib group (RD 0.09, 95% CI 0.07–0.10). This difference was consistent across subgroups (age ≤ or > 75 and lower or higher baseline cardiovascular risk of developing AF). In conclusion, among patients with B-cell malignancies, the risk of developing incident AF is increased when treated with ibrutinib compared to acalabrutinib.

*Trial registration* ClinicalTrial registration number: NCT06561243.

**Supplementary Information:**

The online version contains supplementary material available at 10.1186/s40164-025-00619-6.

## To the editor,

Ibrutinib and acalabrutinib are BTK inhibitors (BTKis) with substantial efficacy for treating B-cell malignancies; however, these drugs are associated with cardiovascular (CV) adverse drug reactions, including notably atrial fibrillation (AF) [1–3]. Consequently, the true incidence and risk of AF associated with BTKis remain unclear [4–6]. The main objective of this study was to investigate the risk of developing incident AF associated with ibrutinib exposure in comparison to acalabrutinib exposure among patients with B-cell malignancy, using real-world data derived from the TriNetX Analytics Network database [7].

The protocol for our study was registered on ClinicalTrials.gov, NCT06561243. Details regarding study oversight, data source and statistical analyses are available in the supplementary data. Data collection and analyses were conducted in July 2024 via the TriNetX online platform. We established a retrospective cohort of adult patients (≥ 18 years) previously diagnosed with a B-cell malignancy (using ICD-10-CM codes) in whom a first BTKi introduction occurred between January 1st, 2013 (first patient exposed to ibrutinib in TriNetX) and July 1st, 2024. Groups (based on ibrutinib or acalabrutinib exposure) were then matched utilizing propensity-score matching (PSM) incorporating 37 covariates, including diagnoses, comorbidities and drugs exposures (supplementary data). Our main objective was to investigate the risk of incident AF in B-cell malignancy patients exposed to ibrutinib compared to those exposed to acalabrutinib in the whole matched cohort. ICD-10-CM code I48 was used to identify the AF outcome (supplementary Tables 1 and 2). Follow-up started from 1 day after first BTKi introduction (patients who experienced AF prior to BTKi introduction were excluded) and continued over a 6-year follow-up period. Post-hoc sensitivity analyses of the primary outcome were conducted to assess the robustness of the results, by recalculating the comparison between the 2 matched groups across several follow-up periods (i) from 1 day after first BTKi introduction and over a 4-year follow-up period, and (ii) from 1 year after first BTKi introduction and over a 4-year follow-up period. Additionally, we assessed the risk of incident AF in the ibrutinib exposed group compared to that in the acalabrutinib-exposed group based on: i. the patient age (≤ 75 and > 75 years) and ii. the baseline cardiovascular risk profiles (low and high risk of developing incident AF; high risk was defined as age ≥ 70 associated with at least one concurrent comorbidity including hypertension, heart failure, diabetes, ischemic stroke or vascular disease). Our secondary objectives were to investigate the risk of all-cause mortality, incident intra-cerebral hemorrhages, major bleedings, hypertension, MACE and a composite of ventricular tachycardia/ventricular fibrillation/cardiac arrest). Cox proportional hazard models were used to calculate the hazard ratios (HRs) and 95% confidence intervals (CIs) to compare the 2 matched groups. The appropriateness of the proportional hazard assumption was examined and risk differences (RDs) were used if appropriate. Results were summarized with the use of Kaplan–Meier survival curves. Statistical analyses were completed using the TriNetX online platform with R for statistical computing. A p-value < 0.05 was deemed statistically significant.

**Fig. 1:**
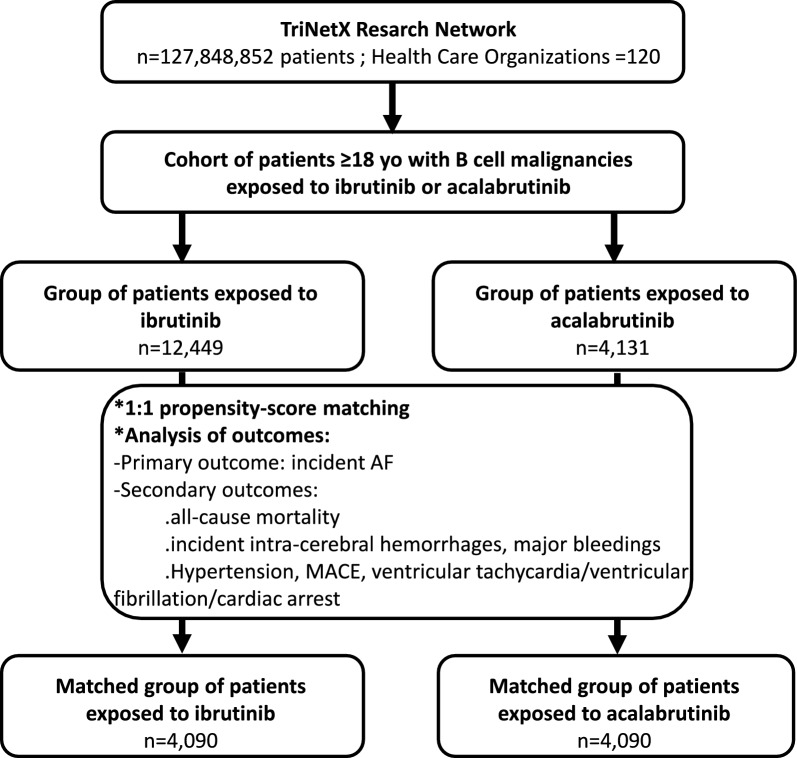
Study consort diagram. Consort diagram depicting inclusion and exclusion criteria. AF means atrial fibrillation

**Fig. 2:**
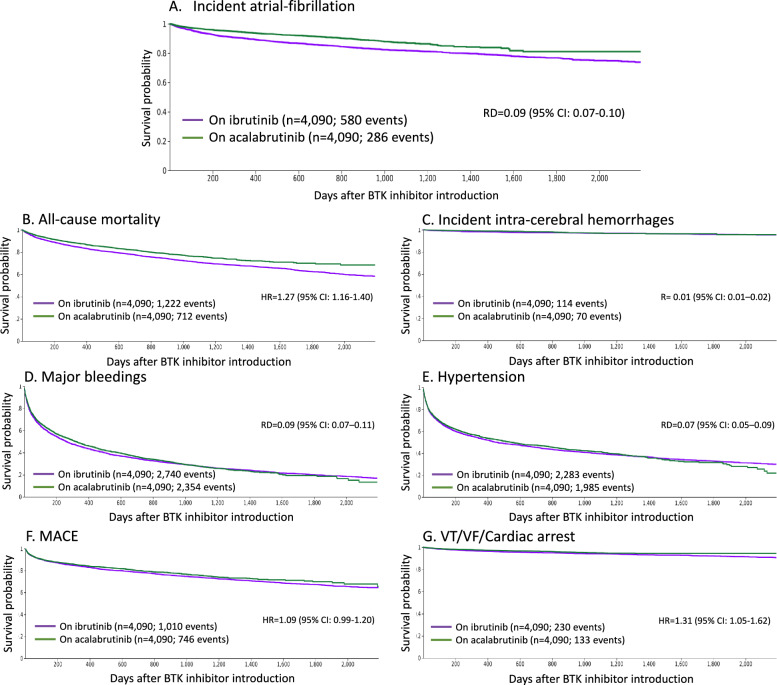
Event-free survival probabilities. **A**: incident atrial fibrillation; **B**: all-cause mortality; **C**: incident intra-cerebral hemorrhages; **D**: major bleedings; panel **E**: hypertension; **F**: MACE (composite of acute myocardial infraction, ischemic stroke or systemic embolism and heart failure) and **G**: Ventricular tachycardia/ventricular fibrillation/cardiac arrest (composite of ventricular tachycardia, ventricular fibrillation and cardiac arrest). Results were summarized with the use of Kaplan–Meier survival curves

Further information on study population, patient characteristics and outcomes is available in the supplementary data and supplementary Table 3. After PSM 4,090 patients remained in each matched group (1:1) and were included in the main analysis (Fig. 1). Treatments on pathway analyses revealed that fewer than 8% of patients switched from one BTKi to another during the follow-up period (supplementary Fig. 1). Over a mean BTKi exposure duration of 2.3 ± 1.8 years (2.9 ± 2.1 and 1.8 ± 1.4 years for ibrutinib and acalabrutinib respectively), we found a higher risk of incident AF in the ibrutinib group compared to the acalabrutinib group in the whole matched cohort (RD 0.09, 95% CI 0.07–0.10; supplementary Table 4 and Fig. 2). Sensitivity analyses were consistent with the main result (supplementary data), and this difference remained consistent across patient aged ≤ 75 and > 75 years as well as in low vs. high-risk patients for developing AF (supplementary Tables 7 and 10). The risk of all-cause mortality was significantly higher in the ibrutinib group when compared to the acalabrutinib group (HR 1.27, 95% CI 1.16–1.40; supplementary Table 4 and Fig. 2). Conversely, no significant differences were observed in the risk of intra-cerebral haemorrhages, major bleedings, hypertension and MACE (Supplementary Table 4 and Fig. 2). The risk of ventricular tachycardia/ventricular fibrillation/cardiac arrest was significantly higher in the ibrutinib-exposed group compared to the acalabrutinib-exposed group (HR 1.31, 95% CI 1.05–1.63; supplementary Table 4 and Fig. 2). Study limitations are available in the supplementary data.

Our study highlights that patients with B-cell malignancies who are exposed to ibrutinib exhibit a significantly increased risk of incident AF in comparison to those exposed to acalabrutinib.

## Supplementary Information

Additional file 1.

## Data Availability

The datasets used and/or analyzed during this study consist of data sourced from the TriNetx platform. Due to TriNetx’s data sharing policies, we do not have access to individual-level data and actually data from TriNetX are only available for health care organizations participating to the health research network database.

## Abbreviations

AF: Atrial fibrillation
BTKis: Bruton's tyrosine kinase inhibitors
CI: Confidence interval
CV: Cardiovascular
HRs: Hazard ratios
ICD-10-CM: International statistical classification of diseases and related health problems, tenth revision, clinical modification
PSM: Propensity-score matching
