# Supplementary material for: Ibrutinib and acalabrutinib use and risk of incident atrial fibrillation: a propensity-matched analysis
Source: Exp Hematol Oncol. 2025 Mar 4;14:29. doi: 10.1186/s40164-025-00619-6 (PMC11877785; doi:10.1186/s40164-025-00619-6)

**Supplementary data 1-** Study oversight, data source and details of statistical analyses.

**Study oversight**

The data were analyzed and interpreted by the authors. All authors reviewed the manuscript and affirmed the accuracy and completeness of the data. The protocol was exempt from institutional review board approval and did not necessitate participant consent given that de-identified data were used from a research network database. The TriNetX platform[1] adheres to standards of the Health Insurance Portability and Accountability Act and the General Data Protection Regulation. The study findings are reported in accordance with the Strengthening the Reporting of Observational Studies in Epidemiology guidelines for cohort studies ([STROBE](https://www.equator-network.org/reporting-guidelines/strobe/)).

**Data source**

We used the TriNetX Analytics Network research database, which represents the world’s largest and most comprehensive real-world clinical data and evidence ecosystem within the life sciences and healthcare domains. TriNetX is an international multicenter federated health research network aggregating anonymized data from electronic health records of more than 120 million patients at the time of our search, sourced from 120 health care organizations (HCOs). These HCOs are grouped into a network known as the Global Collaborative Network and predominantly located in the USA with additional representation in Australia, the UK, Spain, Bulgaria, India, Malaysia, and Taiwan. These HCOs include hospitals, primary care offices, and specialist offices providing data from diverse clinical settings and encompassing both uninsured and insured patients. The TriNetX Analytics Network database contains healthcare data, including demographics, diagnoses represented by the International Statistical Classification of Diseases and Related Health Problems, Tenth Revision, Clinical Modification (ICD-10-CM) codes from outpatient or inpatient, drugs, laboratory values, genomic information and procedures. TriNetX employs a standardized framework to ensure data quality that encompasses conformance, completeness, and plausibility metrics.[2] Data are deidentified per the deidentification standard defined in section §164.514(a) of the Health Insurance Portability and Accountability Act Privacy Rule and are presented as unique patient counts, and a patient is counted only once. The data within TriNetX also exclude patients with only a medical record number or without diagnoses or codes. Although the data are organized in an aggregate de-identified form, the built-in analytics capabilities allow for the generation of patient-level data for cohort selection and matching, analysis of incidence and prevalence of events within a cohort, and comparison of characteristics and outcomes between matched cohorts.[1,3–5] ICD-10-CM codes C91 (lymphoid leukemia), C88.0 (Waldenström macroglobulinemia), C83.1 (mantle cell lymphoma), C81-C96 (malignant neoplasms of lymphoid, hematopoietic and related tissue) or C95 (leukemia of unspecified cell type) were used to identify patients with B-cell malignancies.

**Statistical analyses**

The cohort of patients with B-cell malignancies was stratified into 2 groups based on their initial exposure to either ibrutinib or acalabrutinib. Continuous variables were expressed as mean ± SD and were compared between the 2 groups utilizing independent-sample Student’s t-tests. Categorical variables are reported as n(%) and were compared using the Chi-square test (X²). Covariates were matched extensively using TriNetX’s built-in capacity to generate propensity scores and implement 1:1 matching via greedy nearest neighbor matching. We assessed the comparability between the 2 groups before and after matching using standardized mean differences. The standardized mean difference is a quantitative method used to represent the difference between the means of 2 groups expressed in terms of SD units to assess the balance in measured variables in the sample weighted by the inverse probability of treatment. Any characteristic exhibiting a standardized mean difference of <10% between groups was deemed well matched. No imputations were conducted for missing data. Intra-cerebral hemorrhage outcomes excluded patients with events occurring prior to the specified time window, thereby categorizing these as incident events; other secondary outcomes included patients with events prior to the specified time window and were considered as prevalent events.

**Results**

**Study population and patient characteristics**

A cohort of 16,580 adult patients diagnosed with a B-cell malignancy and exposed to either ibrutinib or acalabrutinib was included in this study. Among these, 12,449 patients had been exposed to ibrutinib while 4,131 had been exposed to acalabrutinib. Treatments on pathway analyses revealed that less than 8% of patients switched from one BTKi to another during the follow-up period (**supplementary Figure 1**). The mean duration of BTKi exposure was 2.3±1.8 years (2.9±2.1 and 1.8±1.4 years for ibrutinib and acalabrutinib respectively, **supplementary Figure 2**).

Before PSM, patients exposed to ibrutinib (compared with those exposed to acalabrutinib) were younger, had a lower prevalence of cardiovascular risk factors, established cardiovascular diseases (except for intracranial hemorrhages) and chronic kidney diseases, and were less likely to be on cardiovascular drugs at baseline. After PSM, baseline characteristics across the 2 groups were comparable with no residual imbalances detected (standardized mean difference <10% for all covariates; **supplementary Table 3** and **supplementary Figure 3**).

The observed higher risk of incident AF in the ibrutinib group compared to the acalabrutinib group seen in our main analysis was consistent across the 2 post-hoc sensitivity analyses (RD 0.07, 95% CI 0.05–0.08 and RD 0.08, 95% CI 0.07–0.09 for i. and ii., respectively).

The baseline characteristics of patients ≤75 or >75 years, before and after PSM, are presented in **supplementary Tables 5 and 6**, respectively. After PSM, baseline characteristics between the 2 groups were comparable with no residual imbalance identified (standardized mean difference <10% for all covariates). We found a higher risk of incident AF in the ibrutinib group compared to the acalabrutinib group in both matched cohorts restricted to patients aged **≤**75 (RD 0.07, 95% CI 0.06–0.09) and restricted to patients aged >75 (RD 0.09, 95% CI 0.07–0.12) without any significant interaction (p for interaction =0.06) (**supplementary Table 7**). Baseline characteristics of patients categorized as having low or high baseline CV risk for developing AF, before and after PSM, are presented in **supplementary Tables 8 and 9**, respectively. After PSM, baseline characteristics were comparable between the 2 groups, except for aortic stenosis, cognitive impairment, hemoglobin A1c and estimated GFR which exhibited a significant residual imbalance between the ibrutinib and acalabrutinib groups (standardized mean difference >10% for these covariates). We found a higher risk of incident AF in the ibrutinib group compared with the acalabrutinib group among both patients with a lower (RD 0.03, 95% CI 0.01–0.05) and those with a higher baseline cardiovascular risk of developing AF (RD 0.11, 95% CI 0.08–0.14) without any significant interaction (p for interaction =0.06) (**supplementary Table 10**).

**Secondary outcomes**

The risk of all-cause mortality was significantly higher in the ibrutinib group compared to the acalabrutinib group (HR 1.27, 95% CI 1.16–1.40; **supplementary table 4** and **Figure 2**). The risks of incident intra-cerebral haemorrhages and major bleedings did not significantly differ between the ibrutinib and acalabrutinib groups (RD 0.01, 95% CI 0.01–0.02 and RD 0.09, 95% CI 0.07–0.11; respectively; **Supplementary Table 4** and **Figure 2**).

The risks of hypertension and MACE did not significantly differ between the ibrutinib and acalabrutinib groups (RD 0.07, 95% CI 0.05–0.09 and HR 1.09, 95% CI 0.99–1.20, respectively; **Supplementary Table 4** and **Figure 2**). The risk of VT/VF/cardiac arrest was significantly higher in the ibrutinib-exposed group compared to the acalabrutinib-exposed group (HR 1.31, 95% CI 1.05–1.63; **Supplementary Table 4** and **Figure 2**).

**Study limitations**

Our study is subject to several limitations. As this study is ground on electronic health records wherein diagnoses and comorbidities identification are based on ICD-10-CM codes, there is a possibility of underreporting, particularly regarding lower grade or asymptomatic events; events such as hypertension may be especially affected by this bias. The absence of ICD-10-CM codes does not necessarily signify the absence of the diagnosis or comorbidity but may be missing data. Consequently, the incidence rates of clinical events reported herein may not accurately reflect real-world incidence rates. Furthermore, detailed quantifications of BTKis dosages and concomitant exposure to other anticancer drugs were not performed. Diagnoses, comorbidities and drugs up to 20 years prior to BTKi introduction were retrieved and thus may not represent conditions ongoing at the time of analysis, potentially introducing bias into the analyses. However, these limitations might equally affect both the ibrutinib and acalabrutinib groups, potentially leading to an unbiased discrepancy between the 2 groups. The first patient exposed to ibrutinib in TriNetX was in 2013 whereas the first patient exposed to acalabrutinib appeared in 2016; this discrepancy may have affected our results due to the evolution of medical practices over this intervening three-year interval. Due to the differing labelling dates for ibrutinib and acalabrutinib, the mean exposure duration between these 2 groups was not similar which could account for the observed lower incidence of AFs in the acalabrutinib group although the median time to onset of cardiac events was recorded at 10.1 months in a pooled analysis of 762 patients exposed to acalabrutinib.[6] It is conceivable that AF diagnoses in this study predominantly correspond to symptomatic and/or severe AF episodes, implying that some cases of asymptomatic AF may not have been captured, and therefore our study is not designed to address the actual real incidence of AF associated with BTKis exposure. Unfortunately, the TriNetX online platform does not permit the identification of the specific countries providing patients to the study. Despite the availability of numerous covariates in the TriNetX database, it was impossible to compute the CHA_2_DS_2_VA score. We selected patients based on a temporal association between exposure to BTKis and the development of AF; however, we cannot definitively attribute causation to BTKis in all cases. It is essential to recognize that the increased risk of all-cause mortality observed in the ibrutinib group may partially be attributable to non-cardiac events that were not addressed in this study. Finally, despite employing PSM using a multitude of covariates to form matched groups with no residual imbalance in our primary analysis, the potential for unaccounted hidden confounders remains.

**References**

1. TriNetX | Connecting the world to improve human health [Internet]. TriNetX. [cited 2024 Jul 10]. Available from: https://trinetx.com/about-trinetx/

2. Kahn MG, Callahan TJ, Barnard J, Bauck AE, Brown J, Davidson BN, et al. A Harmonized Data Quality Assessment Terminology and Framework for the Secondary Use of Electronic Health Record Data. EGEMS (Wash DC). 2016;4:1244.

3. Yen F-S, Wang S-I, Hsu C-C, Hwu C-M, Wei JC-C. Sodium-Glucose Cotransporter-2 Inhibitors and Nephritis Among Patients With Systemic Lupus Erythematosus. JAMA Netw Open. 2024;7:e2416578.

4. Avula V, Sharma G, Kosiborod MN, Vaduganathan M, Neilan TG, Lopez T, et al. SGLT2 Inhibitor Use and Risk of Clinical Events in Patients With Cancer Therapy-Related Cardiac Dysfunction. JACC Heart Fail. 2024;12:67–78.

5. Bucci T, Pastori D, Pignatelli P, Ntaios G, Abdul-Rahim AH, Violi F, et al. Albumin Levels and Risk of Early Cardiovascular Complications After Ischemic Stroke: A Propensity-Matched Analysis of a Global Federated Health Network. Stroke. 2024;55:604–12.

6. Brown JR, Byrd JC, Ghia P, Sharman JP, Hillmen P, Stephens DM, et al. Cardiovascular adverse events in patients with chronic lymphocytic leukemia receiving acalabrutinib monotherapy: pooled analysis of 762 patients. Haematologica. 2022;107:1335–46.

**Supplementary data 2-** Outcomes identified using ICD-10-CM. For outcome definitions consisting of more than one term, at least one term must match.

Propensity score matching was performed on 37 characteristics included diagnoses, comorbidities and drugs exposures up to 20 years ago prior to BTKi introduction. In the Demographics category patients were matched on Age at Index, Male characteristic(s). In the Diagnosis category patients were matched on Essential (primary) hypertension, Chronic ischemic heart disease, Cerebral infarction, Diabetes mellitus, Overweight, obesity and other hyperalimentation, Disorders of thyroid gland, Malnutrition, Disorders of lipoprotein metabolism and other lipidemias, Amyloidosis, Heart failure, Nonrheumatic mitral valve disorders, Atrioventricular and left bundle-branch block, Left bundle-branch block, unspecified, Acute kidney failure and chronic kidney disease, Chronic obstructive pulmonary disease, unspecified, Diseases of the respiratory system, Sleep apnea, Personal history of nicotine dependence, Dilated cardiomyopathy, Atrial fibrillation and flutter, Presence of cardiac pacemaker, Presence of automatic (implantable) cardiac defibrillator, Alcohol abuse, Alzheimer's disease characteristic(s). In the Drugs category patients were matched on BETA BLOCKERS/RELATED, ANTILIPEMIC AGENTS, DIURETICS, ANTIARRHYTHMICS, CALCIUM CHANNEL BLOCKERS, ACE INHIBITORS, ANGIOTENSIN II INHIBITOR, DRUGS USED IN DIABETES, ANTICOAGULANTS, PLATELET AGGREGATION INHIBITORS characteristic(s). In the Laboratory category patients were matched on Blood Pressure, Systolic characteristic(s).

**Supplementary Table 1-** Outcomes identified using ICD-10-CM. For outcome definitions consisting of more than one term, at least one term must match.

| All-cause mortality | | | | |
| --- | --- | --- | --- | --- |
|  | **Outcome definition** | | | |
|  | | Diagnosis | UMLS:ICD10CM:R99 | Ill-defined and unknown cause of mortality |
| Incident intra-cerebral hemorrhage | | | | |
|  | **Outcome definition** | | | |
|  | | Diagnosis | UMLS:ICD10CM:I60 | Nontraumatic subarachnoid hemorrhage |
|  | | Diagnosis | UMLS:ICD10CM:I61 | Nontraumatic intracerebral hemorrhage |
|  | | Diagnosis | UMLS:ICD10CM:I62 | Other and unspecified nontraumatic intracranial hemorrhage |
| Major bleeding | | | | |
|  | **Outcome definition** | | | |
|  | | Diagnosis | UMLS:ICD10CM:I60 | Nontraumatic subarachnoid hemorrhage |
|  | | Diagnosis | UMLS:ICD10CM:I61 | Nontraumatic intracerebral hemorrhage |
|  | | Diagnosis | UMLS:ICD10CM:I62 | Other and unspecified nontraumatic intracranial hemorrhage |
|  | | Diagnosis | UMLS:ICD10CM:J94.2 | Hemothorax |
|  | | Diagnosis | UMLS:ICD10CM:I31.2 | Hemopericardium, not elsewhere classified |
|  | | Diagnosis | UMLS:ICD10CM:M25.0 | Hemarthrosis |
|  | | Diagnosis | UMLS:ICD10CM:N93 | Other abnormal uterine and vaginal bleeding |
|  | | Diagnosis | UMLS:ICD10CM:R31 | Hematuria |
|  | | Diagnosis | UMLS:ICD10CM:R04 | Hemorrhage from respiratory passages |
|  | | Diagnosis | UMLS:ICD10CM:K66 | Other disorders of peritoneum |
|  | | Diagnosis | UMLS:ICD10CM:R50-R69 | General symptoms and signs |
|  | | Diagnosis | UMLS:ICD10CM:K92.1 | Melena |
| Incident atrial fibrillation | | | | |
|  | **Outcome definition** | | | |
|  | | Diagnosis | UMLS:ICD10CM:I48 | Atrial fibrillation and flutter |
| Hypertension | | | | |
|  | **Outcome definition** | | | |
|  | | Diagnosis | UMLS:ICD10CM:I10-I1A | Hypertensive diseases |
| VT/VF/cardiac arrest (composite of ventricular tachycardia, ventricular fibrillation and cardiac arrest) | | | | |
|  | **Outcome definition** | | | |
|  | | Diagnosis | UMLS:ICD10CM:I46 | Cardiac arrest |
|  | | Diagnosis | UMLS:ICD10CM:I49.01 | Ventricular fibrillation |
|  | | Diagnosis | UMLS:ICD10CM:I49.0 | Ventricular fibrillation and flutter |
|  | | Diagnosis | UMLS:ICD10CM:I47.2 | Ventricular tachycardia |
| MACE (composite of acute myocardial infraction, ischemic stroke or systemic embolism and heart failure) | | | | |
|  | **Outcome definition** | | | |
|  | | Diagnosis | UMLS:ICD10CM:I21 | Acute myocardial infarction |
|  | | Diagnosis | UMLS:ICD10CM:I74 | Arterial embolism and thrombosis |
|  | | Diagnosis | UMLS:ICD10CM:I63 | Cerebral infarction |
|  | | Diagnosis | UMLS:ICD10CM:I50 | Heart failure |

**Supplementary Table 2-** Comorbidities identified using ICD-10-CM. For comorbidity definitions consisting of more than one term, at least one term must match.

| **Diagnosis** | |
| --- | --- |
| I10 | Essential (primary) hypertension |
| I25 | Chronic ischemic heart disease |
| I21 | Acute myocardial infarction |
| I63 | Cerebral infarction |
| I61 | Nontraumatic intracerebral hemorrhage |
| E08-E13 | Diabetes mellitus |
| E65-E68 | Overweight, obesity and other hyperalimentation |
| E00-E07 | Disorders of thyroid gland |
| E40-E46 | Malnutrition |
| E78 | Disorders of lipoprotein metabolism and other lipidemias |
| I50 | Heart failure |
| I34 | Nonrheumatic mitral valve disorders |
| N17-N19 | Acute kidney failure and chronic kidney disease |
| J44.9 | Chronic obstructive pulmonary disease, unspecified |
| J00-J99 | Diseases of the respiratory system |
| G47.3 | Sleep apnea |
| I73.9 | Peripheral vascular disease, unspecified |
| Z87.891 | Personal history of nicotine dependence |
| I34.0 | Nonrheumatic mitral (valve) insufficiency |
| I35.0 | Nonrheumatic aortic (valve) stenosis |
| I35.1 | Nonrheumatic aortic (valve) insufficiency |
| I05 | Rheumatic mitral valve diseases |
| I42.0 | Dilated cardiomyopathy |
| I48 | Atrial fibrillation and flutter |
| Z95.0 | Presence of cardiac pacemaker |
| Z95.810 | Presence of automatic (implantable) cardiac defibrillator |
| F10.1 | Alcohol abuse |
| G30 | Alzheimer's disease |
| E10 | Type 1 diabetes mellitus |

**Supplementary Table 3-** Baseline characteristics of all patients before and after propensity score matching in both the ibrutinib and the acalabrutinib exposed groups. Values are mean ± SD or n (%). Any characteristic with a standardized mean difference between groups <10% was considered to be well matched. ICD: implantable cardioverter-defibrillator, COPD: chronic obstructive pulmonary disease, ACE: angiotensin-converting enzyme. Std diff. means standardized mean difference.

|  | **Before propensity-score matching** | | | **After propensity-score matching** | | | | |
| --- | --- | --- | --- | --- | --- | --- | --- | --- |
|  | **Ibrutinib** | **Acalabrutinib** | **Std diff. (%)** | | **Ibrutinib** | **Acalabrutinib** | **Std diff. (%)** |  |
|  | **(n = 12,449)** | **(n = 4,131)** |  |  | **(n = 4,090)** | **(n = 4,090)** |  |  |
| **Demographics** |  |  |  | |  |  |  |  |
| **Age at Index, n (%)** | 67.5 +/- 11.0 | 70.3 +/- 10.0 | 27.2 | | 70.0 +/- 9.8 | 70.3 +/- 10.0 | 2.2 |  |
| **Men, n (%)** | 7,850 (63.1%) | 2,602 (63%) | 0.1 | | 2,578 (63%) | 2,579 (63.1%) | 0.1 |  |
| **Cardiovascular risk factors** |  |  |  | |  |  |  |  |
| **Hypertension, n (%)** | 5,289 (42.5%) | 2,002 (48.5%) | 12 | | 1,894 (46.3%) | 1,969 (48.1%) | 3.7 |  |
| **Systolic BP (mm Hg), mean±SD** | 128.5 +/- 20.2 | 128.2 +/- 19.6 | 1.4 | | 128.5 +/- 20.3 | 128.2 +/- 19.6 | 1.5 |  |
| **Diastolic BP (mm Hg), mean±SD** | 70.9 +/- 11.8 | 69.9 +/- 11.6 | 7.9 | | 70.1 +/- 11.6 | 70 +/- 11.6 | 0.4 |  |
| **Diabetes mellitus, n (%)** | 2,293 (18.4%) | 781 (18.9%) | 1.2 | | 752 (18.4%) | 773 (18.9%) | 1.3 |  |
| **Smoker, n (%)** | 1,758 (14.1%) | 688 (16.7%) | 7 | | 661 (16.2%) | 674 (16.5%) | 0.9 |  |
| **Overweight or obesity, n (%)** | 1,445 (11.6%) | 636 (15.4%) | 11.1 | | 617 (15.1%) | 612 (15%) | 0.3 |  |
| **Dyslipidaemia, n (%)** | 4,331 (34.8%) | 1,775 (43%) | 16.8 | | 1,697 (41.5%) | 1,738 (42.5%) | 2 |  |
| **Cardiovascular comorbidities** |  |  |  | |  |  |  |  |
| **Heart failure, n (%)** | 1,086 (8.7%) | 465 (11.3%) | 8.5 | | 433 (10.6%) | 442 (10.8%) | 0.7 |  |
| **Coronary artery disease, n (%)** | 1,840 (14.8%) | 833 (20.2%) | 14.2 | | 809 (19.8%) | 808 (19.8%) | 0.1 |  |
| **Myocardial infarction, n (%)** | 366 (2.9%) | 166 (4%) | 5.9 | | 143 (3.5%) | 158 (3.9%) | 1.9 |  |
| **Dilated cardiomyopathy, n (%)** | 52 (0.4%) | 49 (1.2%) | 8.6 | | 39 (1%) | 34 (0.8%) | 1.3 |  |
| **Ischemic stroke, n (%)** | 349 (2.8%) | 126 (3.1%) | 1.5 | | 135 (3.3%) | 124 (3%) | 1.5 |  |
| **Intracranial haemorrhage, n (%)** | 84 (0.7%) | 15 (0.4%) | 4.3 | | 29 (0.7%) | 15 (0.4%) | 4.7 |  |
| **Peripheral vascular disease, n (%)** | 367 (2.9%) | 146 (3.5%) | 3.3 | | 152 (3.7%) | 143 (3.5%) | 1.2 |  |
| **Valve disease, n (%)** | 722 (5.8%) | 309 (7.5%) | 6.8 | | 313 (7.7%) | 297 (7.3%) | 1.5 |  |
| **Mitral regurgitation, n (%)** | 662 (5.3%) | 279 (6.8%) | 6 | | 287 (7%) | 267 (6.5%) | 1.9 |  |
| **Mitral stenosis, n (%)** | 85 (0.7%) | 33 (0.8%) | 1.4 | | 35 (0.9%) | 31 (0.8%) | 1.1 |  |
| **Aortic regurgitation, n (%)** | 312 (2.5%) | 129 (3.1%) | 3.7 | | 136 (3.3%) | 122 (3%) | 2 |  |
| **Aortic stenosis, n (%)** | 340 (2.7%) | 143 (3.5%) | 4.2 | | 160 (3.9%) | 132 (3.2%) | 3.7 |  |
| **Atrial fibrillation or flutter, n (%)** | 1,325 (10.6%) | 694 (16.8%) | 18 | | 665 (16.3%) | 663 (16.2%) | 0.1 |  |
| **Previous pacemaker, n (%)** | 136 (1.1%) | 92 (2.2%) | 8.9 | | 76 (1.9%) | 80 (2%) | 0.7 |  |
| **Previous ICD, n (%)** | 55 (0.4%) | 34 (0.8%) | 4.8 | | 31 (0.8%) | 29 (0.7%) | 0.6 |  |
| **Other comorbidities** |  |  |  | |  |  |  |  |
| **Alcohol related diagnoses, n (%)** | 166 (1.3%) | 54 (1.3%) | 0.2 | | 50 (1.2%) | 53 (1.3%) | 0.7 |  |
| **Kidney disease, n (%)** | 2,685 (21.6%) | 1,028 (24.9%) | 7.9 | | 961 (23.5%) | 1,000 (24.4%) | 2.2 |  |
| **Lung disease, n (%)** | 6,033 (48.5%) | 2,113 (51.1%) | 5.4 | | 2,005 (49%) | 2,080 (50.9%) | 3.7 |  |
| **COPD, n (%)** | 884 (7.1%) | 301 (7.3%) | 0.7 | | 299 (7.3%) | 296 (7.2%) | 0.3 |  |
| **Sleep apnoea syndrome, n (%)** | 1,156 (9.3%) | 543 (13.1%) | 12.3 | | 517 (12.6%) | 522 (12.8%) | 0.4 |  |
| **Thyroid diseases, n (%)** | 2,023 (16.3%) | 849 (20.6%) | 11.1 | | 823 (20.1%) | 825 (20.2%) | 0.1 |  |
| **Malnutrition, n (%)** | 904 (7.3%) | 270 (6.5%) | 2.9 | | 252 (6.2%) | 269 (6.6%) | 1.7 |  |
| **Cognitive impairment, n (%)** | 48 (0.4%) | 16 (0.4%) | 0 | | 17 (0.4%) | 16 (0.4%) | 0.4 |  |
| **Laboratory values** |  |  |  | |  |  |  |  |
| **Total cholesterol (mg/dL), mean±SD** | 163.9 +/- 49.6 | 157.4 +/- 46.2 | 13.6 | | 160.4 +/- 50.7 | 157.4 +/- 45.8 | 6.2 |  |
| **LDL cholesterol (mg/dL), mean±SD** | 94.0 +/- 38.0 | 90.3 +/- 37.3 | 9.8 | | 91.3 +/- 37.7 | 90.3 +/- 37.0 | 2.8 |  |
| **HDL cholesterol (mg/dL), mean±SD** | 42.5 +/- 18.6 | 41.6 +/- 17.3 | 5.1 | | 42.1 +/- 18.8 | 41.6 +/- 17.3 | 2.5 |  |
| **Triglyceride (mg/dL), mean±SD** | 140.0 +/- 99.5 | 130.6 +/- 81.5 | 10.4 | | 138.1 +/- 110.5 | 130.7 +/- 81.9 | 7.6 |  |
| **Hemoglobin A1c (%), mean±SD** | 6.2 +/- 1.6 | 6.2 +/- 1.5 | 3.4 | | 6.2 +/- 1.6 | 6.2 +/- 1.5 | 0.5 |  |
| **Estimated GFR (MDRD, ml/min), mean±SD** | 72.5 +/- 27.5 | 68.5 +/- 23.5 | 15.5 | | 69.9 +/- 25.2 | 68.6 +/- 23.6 | 5.4 |  |
| **Drugs** |  |  |  | |  |  |  |  |
| **Beta Blockers, n (%)** | 3,993 (32.1%) | 1,509 (36.5%) | 9.4 | | 1,445 (35.3%) | 1,476 (36.1%) | 1.6 |  |
| **Calcium Channel Blockers, n (%)** | 2,774 (22.3%) | 1,086 (26.3%) | 9.4 | | 1,047 (25.6%) | 1,060 (25.9%) | 0.7 |  |
| **ACE Inhibitors, n (%)** | 2,395 (19.2%) | 806 (19.5%) | 0.7 | | 752 (18.4%) | 795 (19.4%) | 2.7 |  |
| **Angiotensin II Inhibitors, n (%)** | 1,751 (14.1%) | 738 (17.9%) | 10.4 | | 735 (18%) | 715 (17.5%) | 1.3 |  |
| **Digitalis glycosides, n (%)** | 204 (1.6%) | 71 (1.7%) | 0.6 | | 95 (2.3%) | 66 (1.6%) | 5.1 |  |
| **Diuretics, n (%)** | 4,352 (35%) | 1,430 (34.6%) | 0.7 | | 1,322 (32.3%) | 1,405 (34.4%) | 4.3 |  |
| **Lipid lowering drugs, n (%)** | 4,498 (36.1%) | 1,751 (42.4%) | 12.8 | | 1,656 (40.5%) | 1,717 (42%) | 3 |  |
| **Glucose-lowering therapy, n (%)** | 2,870 (23.1%) | 930 (22.5%) | 1.3 | | 890 (21.8%) | 918 (22.4%) | 1.6 |  |
| **Antiplatelet therapy, n (%)** | 3,313 (26.6%) | 1,224 (29.6%) | 6.7 | | 1,171 (28.6%) | 1,198 (29.3%) | 1.5 |  |
| **Anticoagulant, n (%)** | 5,556 (44.6%) | 2,002 (48.5%) | 7.7 | | 1,898 (46.4%) | 1,969 (48.1%) | 3.5 |  |

**Supplementary Table 4 -** Clinical outcomes during follow-up in the matched population.

|  | **Matched group of patients exposed to ibrutinib** | | **Matched group of patients exposed to acalabrutinib** | |  |  |  |  |  |  |  |
| --- | --- | --- | --- | --- | --- | --- | --- | --- | --- | --- | --- |
|  | **(n = 4090)** | | **(n = 4090)** | |  |  |  |  |  |  |  |
|  | **Number of events** | **Yearly rate, %** | **Number of events** | **Yearly rate, %** | **Hazard ratio or risk difference (95% CI)** | **p value** |  |  |  |  |  |
| **Primary outcome** |  |  |  |  |  |  |  |  |  |  |  |
| **Incident atrial fibrillation** | 580 | 4.35 | 286 | 3.15 | RD 0.09 (0.07-0.10) | <0.0001 |  |  |  |  |  |
| **Secondary outcomes** |  |  |  |  |  |  |  |  |  |  |  |
| **All-cause mortality** | 1222 | 6.94 | 712 | 5.27 | HR 1.27 (1.16-1.40) | <0.0001 |  |  |  |  |  |
| **Incident intra-cerebral haemorrhages** | 114 | 0.77 | 70 | 0.74 | RD 0.01 (0.01–0.02) | 0.23 |  |  |  |  |  |
| **Major bleedings** | 2740 | 13.85 | 2354 | 14.47 | RD 0.09 (0.07–0.11) | 0.10 |  |  |  |  |  |
| **Hypertension** | 2283 | 11.68 | 1985 | 13.02 | RD 0.07 (0.05–0.09) | 0.34 |  |  |  |  |  |
| **MACE (composite of acute myocardial infarction, ischemic stroke or systemic embolism and heart failure)** | 1010 | 5.93 | 746 | 5.73 | HR 1.09 (0.99-1.20) | 0.09 |  |  |  |  |  |
| **VT/VF/cardiac arrest (composite of ventricular tachycardia, ventricular fibrillation and cardiac arrest)** | 230 | 1.57 | 133 | 0.93 | HR 1.31 (1.05-1.62) | 0.02 |  |  |  |  |  |

**Supplementary Table 5-** Baseline characteristics of patients ≤75years before and after propensity score matching in both the ibrutinib and the acalabrutinib exposed groups. Values are mean ± SD or n (%). Any characteristic with a standardized mean difference between groups <10% was considered to be well matched. ICD: implantable cardioverter-defibrillator, COPD: chronic obstructive pulmonary disease, ACE: angiotensin-converting enzyme. Std diff. means standardized mean difference.

|  | **Before propensity-score matching** | | | **After propensity-score matching** | | |
| --- | --- | --- | --- | --- | --- | --- |
|  | **Ibrutinib** | **Acalabrutinib** | **Std diff. (%)** | **Ibrutinib** | **Acalabrutinib** | **Std diff. (%)** |
|  | **(n = 6,782)** | **(n = 2,272)** |  | **(n = 2,224)** | **(n = 2,224)** |  |
| **Demographics** |  |  |  |  |  |  |
| **Age at Index, n (%)** | 60.0 +/- 9.0 | 63.3 +/- 7.6 | 39.6 | 63.0 +/- 7.1 | 63.2 +/- 7.6 | 2.1 |
| **Men, n (%)** | 4,393 (64.8%) | 1,478 (65.1%) | 0.6 | 1,452 (65.3%) | 1,442 (64.8%) | 0.9 |
| **Cardiovascular risk factors** |  |  |  |  |  |  |
| **Hypertension, n (%)** | 2,507 (37%) | 947 (41.7%) | 9.7 | 910 (40.9%) | 909 (40.9%) | 0.1 |
| **Systolic BP (mm Hg), mean±SD** | 126.9 +/- 19.3 | 126.6 +/- 18.6 | 1.9 | 127.4 +/- 19.0 | 126.6 +/- 18.5 | 4.4 |
| **Diastolic BP (mm Hg), mean±SD** | 73.0 +/- 11.7 | 72.0 +/- 11.4 | 8.2 | 72.3 +/- 11.3 | 72.1 +/- 11.4 | 2.2 |
| **Diabetes mellitus, n (%)** | 1,167 (17.2%) | 395 (17.4%) | 0.5 | 364 (16.4%) | 381 (17.1%) | 2 |
| **Smoker, n (%)** | 935 (13.8%) | 343 (15.1%) | 3.7 | 322 (14.5%) | 324 (14.6%) | 0.3 |
| **Overweight or obesity, n (%)** | 890 (13.1%) | 378 (16.6%) | 9.9 | 340 (15.3%) | 353 (15.9%) | 1.6 |
| **Dyslipidaemia, n (%)** | 1,961 (28.9%) | 801 (35.3%) | 13.6 | 760 (34.2%) | 765 (34.4%) | 0.5 |
| **Cardiovascular comorbidities** |  |  |  |  |  |  |
| **Heart failure, n (%)** | 458 (6.8%) | 181 (8%) | 4.6 | 164 (7.4%) | 160 (7.2%) | 0.7 |
| **Coronary artery disease, n (%)** | 693 (10.2%) | 324 (14.3%) | 12.4 | 306 (13.8%) | 298 (13.4%) | 1.1 |
| **Myocardial infarction, n (%)** | 167 (2.5%) | 67 (2.9%) | 3 | 58 (2.6%) | 63 (2.8%) | 1.4 |
| **Dilated cardiomyopathy, n (%)** | 20 (0.3%) | 30 (1.3%) | 11.5 | 16 (0.7%) | 12 (0.5%) | 2.3 |
| **Ischemic stroke, n (%)** | 164 (2.4%) | 55 (2.4%) | 0 | 62 (2.8%) | 54 (2.4%) | 2.3 |
| **Intracranial haemorrhage, n (%)** | 49 (0.7%) | 11 (0.5%) | 3.1 | 20 (0.9%) | 11 (0.5%) | 4.9 |
| **Peripheral vascular disease, n (%)** | 133 (2%) | 49 (2.2%) | 1.4 | 53 (2.4%) | 47 (2.1%) | 1.8 |
| **Valve disease, n (%)** | 331 (4.9%) | 134 (5.9%) | 4.5 | 124 (5.6%) | 124 (5.6%) | 0 |
| **Mitral regurgitation, n (%)** | 299 (4.4%) | 120 (5.3%) | 4.1 | 106 (4.8%) | 110 (4.9%) | 0.8 |
| **Mitral stenosis, n (%)** | 27 (0.4%) | 15 (0.7%) | 3.6 | 11 (0.5%) | 14 (0.6%) | 1.8 |
| **Aortic regurgitation, n (%)** | 98 (1.4%) | 36 (1.6%) | 1.1 | 30 (1.3%) | 33 (1.5%) | 1.1 |
| **Aortic stenosis, n (%)** | 78 (1.2%) | 28 (1.2%) | 0.8 | 31 (1.4%) | 28 (1.3%) | 1.2 |
| **Atrial fibrillation or flutter, n (%)** | 447 (6.6%) | 254 (11.2%) | 16.2 | 228 (10.3%) | 226 (10.2%) | 0.3 |
| **Previous pacemaker, n (%)** | 31 (0.5%) | 23 (1%) | 6.5 | 21 (0.9%) | 18 (0.8%) | 1.4 |
| **Previous ICD, n (%)** | 17 (0.3%) | 17 (0.7%) | 7.1 | 11 (0.5%) | 10 (0.4%) | 0.7 |
| **Other comorbidities** |  |  |  |  |  |  |
| **Alcohol related diagnoses, n (%)** | 118 (1.7%) | 39 (1.7%) | 0.2 | 34 (1.5%) | 38 (1.7%) | 1.4 |
| **Kidney disease, n (%)** | 1,391 (20.5%) | 462 (20.3%) | 0.4 | 427 (19.2%) | 442 (19.9%) | 1.7 |
| **Lung disease, n (%)** | 3,243 (47.8%) | 1,089 (47.9%) | 0.2 | 1,029 (46.3%) | 1,058 (47.6%) | 2.6 |
| **COPD, n (%)** | 377 (5.6%) | 120 (5.3%) | 1.2 | 108 (4.9%) | 115 (5.2%) | 1.4 |
| **Sleep apnoea syndrome, n (%)** | 630 (9.3%) | 294 (12.9%) | 11.6 | 278 (12.5%) | 269 (12.1%) | 1.2 |
| **Thyroid diseases, n (%)** | 930 (13.7%) | 393 (17.3%) | 9.9 | 385 (17.3%) | 375 (16.9%) | 1.2 |
| **Malnutrition, n (%)** | 567 (8.4%) | 152 (6.7%) | 6.3 | 134 (6%) | 147 (6.6%) | 2.4 |
| **Cognitive impairment, n (%)** | 10 (0.1%) | 10 (0.4%) | 5.4 | 0 (0%) | 10 (0.4%) | 9.5 |
| **Laboratory values** |  |  |  |  |  |  |
| **Total cholesterol (mg/dL), mean±SD** | 172.1 +/- 52.8 | 163.2 +/- 47.3 | 17.8 | 166.7 +/- 49.3 | 164.0 +/- 47.5 | 5.6 |
| **LDL cholesterol (mg/dL), mean±SD** | 99.9 +/- 39.4 | 94.9 +/- 39.2 | 12.5 | 96.4 +/- 37.6 | 95.7 +/- 39.2 | 1.8 |
| **HDL cholesterol (mg/dL), mean±SD** | 42.8 +/- 19.0 | 41.6 +/- 17.1 | 6.7 | 42.1 +/- 18.0 | 41.6 +/- 17.3 | 2.9 |
| **Triglyceride (mg/dL), mean±SD** | 154.1 +/- 117.0 | 138.3 +/- 93.0 | 14.9 | 146.6 +/- 112.0 | 139.1 +/- 93.7 | 7.3 |
| **Hemoglobin A1c (%), mean±SD** | 6.2 +/- 1.7 | 6.3 +/- 1.6 | 2.2 | 6.2 +/- 1.7 | 6.2 +/- 1.6 | 4.8 |
| **Estimated GFR (MDRD, ml/min), mean±SD** | 77.6 +/- 28.8 | 73.2 +/- 23.5 | 16.6 | 74.3 +/- 24.6 | 73.3 +/- 23.4 | 4.2 |
| **Drugs** |  |  |  |  |  |  |
| **Beta Blockers, n (%)** | 1,846 (27.2%) | 711 (31.3%) | 9 | 675 (30.4%) | 675 (30.4%) | 0 |
| **Calcium Channel Blockers, n (%)** | 1,363 (20.1%) | 496 (21.8%) | 4.3 | 471 (21.2%) | 476 (21.4%) | 0.5 |
| **ACE Inhibitors, n (%)** | 1,223 (18%) | 388 (17.1%) | 2.5 | 398 (17.9%) | 376 (16.9%) | 2.6 |
| **Angiotensin II Inhibitors, n (%)** | 787 (11.6%) | 317 (14%) | 7 | 291 (13.1%) | 299 (13.4%) | 1.1 |
| **Digitalis glycosides, n (%)** | 63 (0.9%) | 27 (1.2%) | 2.5 | 27 (1.2%) | 21 (0.9%) | 2.6 |
| **Diuretics, n (%)** | 2,231 (32.9%) | 669 (29.4%) | 7.5 | 642 (28.9%) | 646 (29%) | 0.4 |
| **Lipid lowering drugs, n (%)** | 1,987 (29.3%) | 798 (35.1%) | 12.5 | 762 (34.3%) | 762 (34.3%) | 0 |
| **Glucose-lowering therapy, n (%)** | 1,579 (23.3%) | 511 (22.5%) | 1.9 | 470 (21.1%) | 495 (22.3%) | 2.7 |
| **Antiplatelet therapy, n (%)** | 1,406 (20.7%) | 541 (23.8%) | 7.4 | 500 (22.5%) | 515 (23.2%) | 1.6 |
| **Anticoagulant, n (%)** | 2,990 (44.1%) | 1,009 (44.4%) | 0.7 | 949 (42.7%) | 973 (43.8%) | 2.2 |

**Supplementary Table 6-** Baseline characteristics of patients >75years before and after propensity score matching in both the ibrutinib and the acalabrutinib exposed groups. Values are mean ± SD or n (%). Any characteristic with a standardized mean difference between groups <10% was considered to be well matched. ICD: implantable cardioverter-defibrillator, COPD: chronic obstructive pulmonary disease, ACE: angiotensin-converting enzyme. Std diff. means standardized mean difference.

|  | **Before propensity-score matching** | | | **After propensity-score matching** | | |
| --- | --- | --- | --- | --- | --- | --- |
|  | **Ibrutinib** | **Acalabrutinib** | **Std diff. (%)** | **Ibrutinib** | **Acalabrutinib** | **Std diff. (%)** |
|  | **(n = 6,421)** | **(n = 2,154)** |  | **(n = 2,114)** | **(n = 2,114)** |  |
| **Demographics** |  |  |  |  |  |  |
| **Age at Index, n (%)** | 75.8 +/- 5.0 | 78.4 +/- 4.6 | 53.7 | 78.4 +/- 4.7 | 78.3 +/- 4.5 | 2.8 |
| **Men, n (%)** | 3,918 (61%) | 1,305 (60.6%) | 0.9 | 1,284 (60.7%) | 1,282 (60.6%) | 0.2 |
| **Cardiovascular risk factors** |  |  |  |  |  |  |
| **Hypertension, n (%)** | 3,120 (48.6%) | 1,207 (56%) | 14.9 | 1,119 (52.9%) | 1,176 (55.6%) | 5.4 |
| **Systolic BP (mm Hg), mean±SD** | 130.4 +/- 21.0 | 130.1 +/- 20.7 | 1.4 | 129.4 +/- 20.9 | 130.3 +/- 20.7 | 4 |
| **Diastolic BP (mm Hg), mean±SD** | 68.5 +/- 11.3 | 67.4 +/- 11.3 | 10 | 67.3 +/- 10.9 | 67.5 +/- 11.4 | 1.5 |
| **Diabetes mellitus, n (%)** | 1,271 (19.8%) | 454 (21.1%) | 3.2 | 431 (20.4%) | 444 (21%) | 1.5 |
| **Smoker, n (%)** | 888 (13.8%) | 380 (17.6%) | 10.5 | 359 (17%) | 370 (17.5%) | 1.4 |
| **Overweight or obesity, n (%)** | 636 (9.9%) | 296 (13.7%) | 11.9 | 270 (12.8%) | 282 (13.3%) | 1.7 |
| **Dyslipidaemia, n (%)** | 2,659 (41.4%) | 1,115 (51.8%) | 20.9 | 1,029 (48.7%) | 1,082 (51.2%) | 5 |
| **Cardiovascular comorbidities** |  |  |  |  |  |  |
| **Heart failure, n (%)** | 683 (10.6%) | 319 (14.8%) | 12.5 | 310 (14.7%) | 305 (14.4%) | 0.7 |
| **Coronary artery disease, n (%)** | 1,275 (19.9%) | 579 (26.9%) | 16.7 | 549 (26%) | 561 (26.5%) | 1.3 |
| **Myocardial infarction, n (%)** | 221 (3.4%) | 114 (5.3%) | 9.1 | 99 (4.7%) | 110 (5.2%) | 2.4 |
| **Dilated cardiomyopathy, n (%)** | 32 (0.5%) | 25 (1.2%) | 7.3 | 22 (1%) | 20 (0.9%) | 1 |
| **Ischemic stroke, n (%)** | 209 (3.3%) | 85 (3.9%) | 3.7 | 88 (4.2%) | 81 (3.8%) | 1.7 |
| **Intracranial haemorrhage, n (%)** | 38 (0.6%) | 10 (0.5%) | 1.8 | 17 (0.8%) | 10 (0.5%) | 4.2 |
| **Peripheral vascular disease, n (%)** | 256 (4%) | 105 (4.9%) | 4.3 | 103 (4.9%) | 99 (4.7%) | 0.9 |
| **Valve disease, n (%)** | 421 (6.6%) | 192 (8.9%) | 8.8 | 173 (8.2%) | 186 (8.8%) | 2.2 |
| **Mitral regurgitation, n (%)** | 391 (6.1%) | 175 (8.1%) | 7.9 | 159 (7.5%) | 169 (8%) | 1.8 |
| **Mitral stenosis, n (%)** | 61 (1%) | 20 (0.9%) | 0.2 | 26 (1.2%) | 17 (0.8%) | 4.2 |
| **Aortic regurgitation, n (%)** | 233 (3.6%) | 104 (4.8%) | 6 | 101 (4.8%) | 97 (4.6%) | 0.9 |
| **Aortic stenosis, n (%)** | 282 (4.4%) | 123 (5.7%) | 6 | 128 (6.1%) | 117 (5.5%) | 2.2 |
| **Atrial fibrillation or flutter, n (%)** | 949 (14.8%) | 497 (23.1%) | 21.3 | 473 (22.4%) | 475 (22.5%) | 0.2 |
| **Previous pacemaker, n (%)** | 111 (1.7%) | 79 (3.7%) | 12 | 61 (2.9%) | 70 (3.3%) | 2.5 |
| **Previous ICD, n (%)** | 44 (0.7%) | 23 (1.1%) | 4.1 | 16 (0.8%) | 20 (0.9%) | 2.1 |
| **Other comorbidities** |  |  |  |  |  |  |
| **Alcohol related diagnoses, n (%)** | 54 (0.8%) | 19 (0.9%) | 0.4 | 20 (0.9%) | 18 (0.9%) | 1 |
| **Kidney disease, n (%)** | 1,453 (22.6%) | 645 (29.9%) | 16.7 | 605 (28.6%) | 617 (29.2%) | 1.3 |
| **Lung disease, n (%)** | 3,156 (49.2%) | 1,203 (55.8%) | 13.4 | 1,122 (53.1%) | 1,171 (55.4%) | 4.7 |
| **COPD, n (%)** | 557 (8.7%) | 209 (9.7%) | 3.6 | 200 (9.5%) | 207 (9.8%) | 1.1 |
| **Sleep apnoea syndrome, n (%)** | 596 (9.3%) | 277 (12.9%) | 11.4 | 271 (12.8%) | 264 (12.5%) | 1 |
| **Thyroid diseases, n (%)** | 1,217 (19%) | 525 (24.4%) | 13.2 | 498 (23.6%) | 505 (23.9%) | 0.8 |
| **Malnutrition, n (%)** | 383 (6%) | 139 (6.5%) | 2 | 146 (6.9%) | 137 (6.5%) | 1.7 |
| **Cognitive impairment, n (%)** | 45 (0.7%) | 15 (0.7%) | 0.1 | 18 (0.9%) | 15 (0.7%) | 1.6 |
| **Laboratory values** |  |  |  |  |  |  |
| **Total cholesterol (mg/dL), mean±SD** | 155.8 +/- 45.8 | 151.2 +/- 43.6 | 10.3 | 151.6 +/- 44.8 | 151.5 +/- 43.7 | 0.2 |
| **LDL cholesterol (mg/dL), mean±SD** | 88.3 +/- 35.9 | 84.8 +/- 34.6 | 9.8 | 85.4 +/- 35.7 | 85.0 +/- 34.8 | 1.2 |
| **HDL cholesterol (mg/dL), mean±SD** | 42.4 +/- 18.0 | 42.2 +/- 17.4 | 1 | 41.9 +/- 17.3 | 42.2 +/- 17.2 | 2.1 |
| **Triglyceride (mg/dL), mean±SD** | 124.7 +/- 72.7 | 123.1 +/- 66.3 | 2.4 | 123.0 +/- 68.5 | 123.2 +/- 66.3 | 0.2 |
| **Hemoglobin A1c (%), mean±SD** | 6.2 +/- 1.4 | 6.1 +/- 1.3 | 7.7 | 6.2 +/- 1.5 | 6.1 +/- 1.3 | 5.6 |
| **Estimated GFR (MDRD, ml/min), mean±SD** | 66.5 +/- 24.5 | 63.1 +/- 22.5 | 14.5 | 64.2 +/- 24.8 | 63.3 +/- 22.4 | 3.8 |
| **Drugs** |  |  |  |  |  |  |
| **Beta Blockers, n (%)** | 2,387 (37.2%) | 925 (42.9%) | 11.8 | 870 (41.2%) | 902 (42.7%) | 3.1 |
| **Calcium Channel Blockers, n (%)** | 1,566 (24.4%) | 675 (31.3%) | 15.5 | 628 (29.7%) | 649 (30.7%) | 2.2 |
| **ACE Inhibitors, n (%)** | 1,325 (20.6%) | 467 (21.7%) | 2.6 | 428 (20.2%) | 457 (21.6%) | 3.4 |
| **Angiotensin II Inhibitors, n (%)** | 1,070 (16.7%) | 469 (21.8%) | 13 | 409 (19.3%) | 450 (21.3%) | 4.8 |
| **Digitalis glycosides, n (%)** | 151 (2.4%) | 55 (2.6%) | 1.3 | 66 (3.1%) | 52 (2.5%) | 4 |
| **Diuretics, n (%)** | 2,388 (37.2%) | 875 (40.6%) | 7 | 787 (37.2%) | 847 (40.1%) | 5.8 |
| **Lipid lowering drugs, n (%)** | 2,774 (43.2%) | 1,083 (50.3%) | 14.2 | 1,009 (47.7%) | 1,054 (49.9%) | 4.3 |
| **Glucose-lowering therapy, n (%)** | 1,455 (22.7%) | 497 (23.1%) | 1 | 479 (22.7%) | 483 (22.8%) | 0.5 |
| **Antiplatelet therapy, n (%)** | 2,142 (33.4%) | 798 (37%) | 7.7 | 803 (38%) | 778 (36.8%) | 2.4 |
| **Anticoagulant, n (%)** | 2,914 (45.4%) | 1,137 (52.8%) | 14.8 | 1,068 (50.5%) | 1,105 (52.3%) | 3.5 |

**Supplementary Table 7-** Risk of incident AF in the ibrutinib exposed group compared to the acalabrutinib exposed group in matched-patients ≤75years compared to matched-patients >75years.

|  | **Ibrutinib** | | **Acalabrutinib** | |  |  |
| --- | --- | --- | --- | --- | --- | --- |
|  | **Number of events** | **Yearly rate, %** | **Number of events** | **Yearly rate, %** | **Risk difference  (95% CI)** | **p value** |
| **Matched-patients ≤75years** | **n=2224** | | **n=2224** | |  |  |
| **Incident atrial fibrillation** | 263 | 3.2 | 115 | 1.9 | 0.07 (0.06-0.09) | <0.0001 |
| **Matched-patients >75years** | **n=2114** | | **n=2114** | |  |  |
| **Incident atrial fibrillation** | 344 | 5.7 | 192 | 4.7 | 0.09 (0.07-0.12) | 0.001 |

p for interaction for the risk of AF in patients ≤75 years versus >75 years =0.06

**Supplementary Table 8-** Baseline characteristics of patients with a lower baseline cardiovascular risk of developing AF, before and after propensity score matching in both the ibrutinib and the acalabrutinib exposed groups. Values are mean ± SD or n (%). Any characteristic with a standardized mean difference between groups <10% was considered to be well matched. ICD: implantable cardioverter-defibrillator, COPD: chronic obstructive pulmonary disease, ACE: angiotensin-converting enzyme. Std diff. means standardized mean difference.

|  | **Before propensity-score matching** | | | **After propensity-score matching** | | |
| --- | --- | --- | --- | --- | --- | --- |
|  | **Ibrutinib** | **Acalabrutinib** | **Std diff. (%)** | **Ibrutinib** | **Acalabrutinib** | **Std diff. (%)** |
|  | **(n = 1,949)** | **(n = 722)** |  | **(n = 714)** | **(n = 714)** |  |
| **Demographics** |  |  |  |  |  |  |
| **Age at Index, n (%)** | 56.5 +/- 9.1 | 58.5 +/- 7.7 | 24.5 | 58.6 +/- 7.6 | 58.5 +/- 7.7 | 0.5 |
| **Men, n (%)** | 1,225 (62.9%) | 448 (62%) | 1.7 | 463 (64.8%) | 444 (62.2%) | 5.5 |
| **Cardiovascular risk factors** |  |  |  |  |  |  |
| **Hypertension, n (%)** | 0 (0%) | 0 (0%) | 0 | 0 (0%) | 0 (0%) | 0 |
| **Systolic BP (mm Hg), mean±SD** | 122.0 +/- 18.0 | 124.5 +/- 17.7 | 14.2 | 124.7 +/- 18.5 | 124.5 +/- 17.8 | 1.1 |
| **Diastolic BP (mm Hg), mean±SD** | 71.7 +/- 11.1 | 73.0 +/- 10.8 | 11.6 | 72.2 +/- 10.9 | 73.0 +/- 10.9 | 7.8 |
| **Diabetes mellitus, n (%)** | 0 (0%) | 0 (0%) | 0 | 0 (0%) | 0 (0%) | 0 |
| **Smoker, n (%)** | 143 (7.3%) | 48 (6.6%) | 2.7 | 44 (6.2%) | 47 (6.6%) | 1.7 |
| **Overweight or obesity, n (%)** | 84 (4.3%) | 39 (5.4%) | 5.1 | 36 (5%) | 36 (5%) | 0 |
| **Dyslipidaemia, n (%)** | 221 (11.3%) | 102 (14.1%) | 8.4 | 87 (12.2%) | 99 (13.9%) | 5 |
| **Cardiovascular comorbidities** |  |  |  |  |  |  |
| **Heart failure, n (%)** | 0 (0%) | 0 (0%) | 0 | 0 (0%) | 0 (0%) | 0 |
| **Coronary artery disease, n (%)** | 40 (2.1%) | 13 (1.8%) | 1.8 | 10 (1.4%) | 13 (1.8%) | 3.3 |
| **Myocardial infarction, n (%)** | 10 (0.5%) | 10 (1.4%) | 9 | 10 (1.4%) | 10 (1.4%) | 0 |
| **Dilated cardiomyopathy, n (%)** | 0 (0%) | 0 (0%) | 0 | 0 (0%) | 0 (0%) | 0 |
| **Ischemic stroke, n (%)** | 0 (0%) | 0 (0%) | 0 | 0 (0%) | 0 (0%) | 0 |
| **Intracranial hemorrhage, n (%)** | 10 (0.5%) | 10 (1.4%) | 9 | 10 (1.4%) | 10 (1.4%) | 0 |
| **Peripheral vascular disease, n (%)** | 0 (0%) | 0 (0%) | 0 | 0 (0%) | 0 (0%) | 0 |
| **Valve disease, n (%)** | 35 (1.8%) | 13 (1.8%) | 0 | 10 (1.4%) | 13 (1.8%) | 3.3 |
| **Mitral regurgitation, n (%)** | 32 (1.6%) | 12 (1.7%) | 0.2 | 10 (1.4%) | 12 (1.7%) | 2.3 |
| **Mitral stenosis, n (%)** | 10 (0.5%) | 10 (1.4%) | 9 | 10 (1.4%) | 10 (1.4%) | 0 |
| **Aortic regurgitation, n (%)** | 10 (0.5%) | 10 (1.4%) | 9 | 10 (1.4%) | 10 (1.4%) | 0 |
| **Aortic stenosis, n (%)** | 10 (0.5%) | 0 (0%) | 10.2 | 10 (1.4%) | 0 (0%) | 16.9 |
| **Atrial fibrillation or flutter, n (%)** | 45 (2.3%) | 20 (2.8%) | 2.9 | 21 (2.9%) | 18 (2.5%) | 2.6 |
| **Previous pacemaker, n (%)** | 10 (0.5%) | 0 (0%) | 10.2 | 0 (0%) | 0 (0%) | 0 |
| **Previous ICD, n (%)** | 0 (0%) | 0 (0%) | 0 | 0 (0%) | 0 (0%) | 0 |
| **Other comorbidities** |  |  |  |  |  |  |
| **Alcohol related diagnoses, n (%)** | 16 (0.8%) | 10 (1.4%) | 5.4 | 10 (1.4%) | 10 (1.4%) | 0 |
| **Kidney disease, n (%)** | 199 (10.2%) | 54 (7.5%) | 9.6 | 48 (6.7%) | 52 (7.3%) | 2.2 |
| **Lung disease, n (%)** | 695 (35.7%) | 235 (32.5%) | 6.6 | 221 (31%) | 229 (32.1%) | 2.4 |
| **COPD, n (%)** | 38 (1.9%) | 10 (1.4%) | 4.4 | 10 (1.4%) | 10 (1.4%) | 0 |
| **Sleep apnoea syndrome, n (%)** | 71 (3.6%) | 29 (4%) | 1.9 | 28 (3.9%) | 27 (3.8%) | 0.7 |
| **Thyroid diseases, n (%)** | 174 (8.9%) | 77 (10.7%) | 5.8 | 65 (9.1%) | 74 (10.4%) | 4.3 |
| **Malnutrition, n (%)** | 97 (5%) | 19 (2.6%) | 12.3 | 18 (2.5%) | 19 (2.7%) | 0.9 |
| **Cognitive impairment, n (%)** | 10 (0.5%) | 0 (0%) | 10.2 | 10 (1.4%) | 0 (0%) | 16.9 |
| **Laboratory values** |  |  |  |  |  |  |
| **Total cholesterol (mg/dL), mean±SD** | 178.5 +/- 52.7 | 176.9 +/- 43.3 | 3.5 | 174.2 +/- 47.6 | 176.5 +/- 43.6 | 5.2 |
| **LDL cholesterol (mg/dL), mean±SD** | 106.3 +/- 37.2 | 106.7 +/- 31.1 | 1.4 | 106.1 +/- 37.0 | 106.3 +/- 31.2 | 0.7 |
| **HDL cholesterol (mg/dL), mean±SD** | 44.6 +/- 20.8 | 44.4 +/- 17.8 | 1 | 43.9 +/- 20.9 | 44.3 +/- 18.2 | 2.4 |
| **Triglyceride (mg/dL), mean±SD** | 143.9 +/- 101.5 | 125.2 +/- 87.5 | 19.7 | 117.9 +/- 68.2 | 125.3 +/- 88.4 | 9.4 |
| **Hemoglobin A1c (%), mean±SD** | 5.7 +/- 1.4 | 5.6 +/- 1.0 | 7.6 | 5.8 +/- 2.0 | 5.6 +/- 1.0 | 11.2 |
| **Estimated GFR (MDRD, ml/min), mean±SD** | 80.6 +/- 27.6 | 76.1 +/- 20.7 | 18.8 | 78.4 +/- 24.6 | 76.1 +/- 20.8 | 10.1 |
| **Drugs** |  |  |  |  |  |  |
| **Beta Blockers, n (%)** | 224 (11.5%) | 63 (8.7%) | 9.2 | 61 (8.5%) | 61 (8.5%) | 0 |
| **Calcium Channel Blockers, n (%)** | 97 (5%) | 35 (4.8%) | 0.6 | 36 (5%) | 34 (4.8%) | 1.3 |
| **ACE Inhibitors, n (%)** | 64 (3.3%) | 16 (2.2%) | 6.5 | 21 (2.9%) | 16 (2.2%) | 4.4 |
| **Angiotensin II Inhibitors, n (%)** | 46 (2.4%) | 17 (2.4%) | 0 | 11 (1.5%) | 17 (2.4%) | 6.1 |
| **Digitalis glycosides, n (%)** | 10 (0.5%) | 10 (1.4%) | 9 | 10 (1.4%) | 10 (1.4%) | 0 |
| **Diuretics, n (%)** | 328 (16.8%) | 77 (10.7%) | 18 | 73 (10.2%) | 77 (10.8%) | 1.8 |
| **Lipid lowering drugs, n (%)** | 252 (12.9%) | 97 (13.4%) | 1.5 | 85 (11.9%) | 96 (13.4%) | 4.6 |
| **Glucose-lowering therapy, n (%)** | 155 (8%) | 48 (6.6%) | 5 | 47 (6.6%) | 47 (6.6%) | 0 |
| **Antiplatelet therapy, n (%)** | 188 (9.6%) | 78 (10.8%) | 3.8 | 68 (9.5%) | 74 (10.4%) | 2.8 |
| **Anticoagulant, n (%)** | 653 (33.5%) | 203 (28.1%) | 11.7 | 211 (29.6%) | 201 (28.2%) | 3.1 |

**Supplementary Table 9-** Baseline characteristics of patients with a higher baseline cardiovascular risk of developing AF, before and after propensity score matching in both the ibrutinib and the acalabrutinib exposed groups. Values are mean ± SD or n (%). Any characteristic with a standardized mean difference between groups <10% was considered to be well matched. ICD: implantable cardioverter-defibrillator, COPD: chronic obstructive pulmonary disease, ACE: angiotensin-converting enzyme. Std diff. means standardized mean difference.

|  | **Before propensity-score matching** | | | **After propensity-score matching** | | |
| --- | --- | --- | --- | --- | --- | --- |
|  | **Ibrutinib** | **Acalabrutinib** | **Std diff. (%)** | **Ibrutinib** | **Acalabrutinib** | **Std diff. (%)** |
|  | **(n = 5,908)** | **(n = 2,096)** |  | **(n = 2,061)** | **(n = 2,061)** |  |
| **Demographics** |  |  |  |  |  |  |
| **Age at Index, n (%)** | 74.6 +/- 5.7 | 76.7 +/- 5.5 | 39 | 76.6 +/- 5.5 | 76.7 +/- 5.5 | 0.5 |
| **Men, n (%)** | 3,685 (62.4%) | 1,335 (63.7%) | 2.7 | 1,286 (62.4%) | 1,312 (63.7%) | 2.6 |
| **Cardiovascular risk factors** |  |  |  |  |  |  |
| **Hypertension, n (%)** | 3,683 (62.3%) | 1,518 (72.4%) | 21.6 | 1,463 (71%) | 1,486 (72.1%) | 2.5 |
| **Systolic BP (mm Hg), mean±SD** | 131.5 +/- 20.9 | 130.6 +/- 20.8 | 4.2 | 130.8 +/- 20.5 | 130.7 +/- 20.9 | 0.1 |
| **Diastolic BP (mm Hg), mean±SD** | 69.2 +/- 11.5 | 67.6 +/- 11.2 | 13.9 | 68.6 +/- 11.3 | 67.7 +/- 11.2 | 8.3 |
| **Diabetes mellitus, n (%)** | 1,530 (25.9%) | 569 (27.1%) | 2.8 | 563 (27.3%) | 558 (27.1%) | 0.5 |
| **Smoker, n (%)** | 962 (16.3%) | 425 (20.3%) | 10.3 | 423 (20.5%) | 409 (19.8%) | 1.7 |
| **Overweight or obesity, n (%)** | 754 (12.8%) | 370 (17.7%) | 13.7 | 344 (16.7%) | 352 (17.1%) | 1 |
| **Dyslipidaemia, n (%)** | 2,863 (48.5%) | 1,232 (58.8%) | 20.8 | 1,181 (57.3%) | 1,200 (58.2%) | 1.9 |
| **Cardiovascular comorbidities** |  |  |  |  |  |  |
| **Heart failure, n (%)** | 768 (13%) | 375 (17.9%) | 13.6 | 351 (17%) | 353 (17.1%) | 0.3 |
| **Coronary artery disease, n (%)** | 1,346 (22.8%) | 646 (30.8%) | 18.2 | 626 (30.4%) | 624 (30.3%) | 0.2 |
| **Myocardial infarction, n (%)** | 236 (4%) | 125 (6%) | 9.1 | 103 (5%) | 122 (5.9%) | 4.1 |
| **Dilated cardiomyopathy, n (%)** | 35 (0.6%) | 35 (1.7%) | 10.2 | 28 (1.4%) | 21 (1%) | 3.1 |
| **Ischemic stroke, n (%)** | 246 (4.2%) | 104 (5%) | 3.8 | 110 (5.3%) | 100 (4.9%) | 2.2 |
| **Intracranial hemorrhage, n (%)** | 45 (0.8%) | 10 (0.5%) | 3.6 | 16 (0.8%) | 10 (0.5%) | 3.7 |
| **Peripheral vascular disease, n (%)** | 294 (5%) | 127 (6.1%) | 4.7 | 129 (6.3%) | 126 (6.1%) | 0.6 |
| **Valve disease, n (%)** | 436 (7.4%) | 205 (9.8%) | 8.6 | 189 (9.2%) | 195 (9.5%) | 1 |
| **Mitral regurgitation, n (%)** | 403 (6.8%) | 186 (8.9%) | 7.6 | 176 (8.5%) | 176 (8.5%) | 0 |
| **Mitral stenosis, n (%)** | 65 (1.1%) | 24 (1.1%) | 0.4 | 28 (1.4%) | 22 (1.1%) | 2.7 |
| **Aortic regurgitation, n (%)** | 230 (3.9%) | 102 (4.9%) | 4.8 | 107 (5.2%) | 94 (4.6%) | 2.9 |
| **Aortic stenosis, n (%)** | 282 (4.8%) | 127 (6.1%) | 5.7 | 133 (6.5%) | 120 (5.8%) | 2.6 |
| **Atrial fibrillation or flutter, n (%)** | 962 (16.3%) | 533 (25.4%) | 22.7 | 503 (24.4%) | 509 (24.7%) | 0.7 |
| **Previous pacemaker, n (%)** | 110 (1.9%) | 80 (3.8%) | 11.8 | 67 (3.3%) | 68 (3.3%) | 0.3 |
| **Previous ICD, n (%)** | 44 (0.7%) | 28 (1.3%) | 5.8 | 26 (1.3%) | 20 (1%) | 2.8 |
| **Other comorbidities** |  |  |  |  |  |  |
| **Alcohol related diagnoses, n (%)** | 73 (1.2%) | 26 (1.2%) | 0 | 30 (1.5%) | 26 (1.3%) | 1.7 |
| **Kidney disease, n (%)** | 1,536 (26%) | 717 (34.2%) | 18 | 664 (32.2%) | 691 (33.5%) | 2.8 |
| **Lung disease, n (%)** | 3,157 (53.4%) | 1,281 (61.1%) | 15.6 | 1,225 (59.4%) | 1,251 (60.7%) | 2.6 |
| **COPD, n (%)** | 582 (9.9%) | 233 (11.1%) | 4.1 | 232 (11.3%) | 225 (10.9%) | 1.1 |
| **Sleep apnoea syndrome, n (%)** | 674 (11.4%) | 326 (15.6%) | 12.2 | 310 (15%) | 309 (15%) | 0.1 |
| **Thyroid diseases, n (%)** | 1,203 (20.4%) | 555 (26.5%) | 14.5 | 543 (26.3%) | 532 (25.8%) | 1.2 |
| **Malnutrition, n (%)** | 378 (6.4%) | 152 (7.3%) | 3.4 | 145 (7%) | 149 (7.2%) | 0.8 |
| **Cognitive impairment, n (%)** | 44 (0.7%) | 15 (0.7%) | 0.3 | 15 (0.7%) | 15 (0.7%) | 0 |
| **Laboratory values** |  |  |  |  |  |  |
| **Total cholesterol (mg/dL), mean±SD** | 156.3 +/- 46.1 | 151.0 +/- 43.9 | 11.6 | 152.0 +/- 45.4 | 151.4 +/- 44.2 | 1.3 |
| **LDL cholesterol (mg/dL), mean±SD** | 88.4 +/- 36.2 | 84.6 +/- 35.5 | 10.6 | 84.7 +/- 36.5 | 84.8 +/- 35.7 | 0 |
| **HDL cholesterol (mg/dL), mean±SD** | 42.3 +/- 17.6 | 41.6 +/- 17.0 | 3.8 | 41.5 +/- 16.9 | 41.6 +/- 17.1 | 0.8 |
| **Triglyceride (mg/dL), mean±SD** | 127.5 +/- 76.9 | 127.1 +/- 72.9 | 0.5 | 128.7 +/- 74.6 | 127.7 +/- 73.4 | 1.3 |
| **Hemoglobin A1c (%), mean±SD** | 6.3 +/- 1.5 | 6.2 +/- 1.4 | 4.3 | 6.3 +/- 1.5 | 6.2 +/- 1.4 | 4.9 |
| **Estimated GFR (MDRD, ml/min), mean±SD** | 66.2 +/- 24.6 | 63.4 +/- 23.2 | 11.4 | 63.5 +/- 24.2 | 63.6 +/- 23.2 | 0.5 |
| **Drugs** |  |  |  |  |  |  |
| **Beta Blockers, n (%)** | 2,470 (41.8%) | 1,029 (49.1%) | 14.7 | 993 (48.2%) | 1,001 (48.6%) | 0.8 |
| **Calcium Channel Blockers, n (%)** | 1,725 (29.2%) | 764 (36.5%) | 15.5 | 724 (35.1%) | 741 (36%) | 1.7 |
| **ACE Inhibitors, n (%)** | 1,494 (25.3%) | 562 (26.8%) | 3.5 | 543 (26.3%) | 549 (26.6%) | 0.7 |
| **Angiotensin II Inhibitors, n (%)** | 1,204 (20.4%) | 540 (25.8%) | 12.8 | 521 (25.3%) | 521 (25.3%) | 0 |
| **Digitalis glycosides, n (%)** | 142 (2.4%) | 54 (2.6%) | 1.1 | 68 (3.3%) | 46 (2.2%) | 6.5 |
| **Diuretics, n (%)** | 2,479 (42%) | 972 (46.4%) | 8.9 | 916 (44.4%) | 946 (45.9%) | 2.9 |
| **Lipid lowering drugs, n (%)** | 2,836 (48%) | 1,165 (55.6%) | 15.2 | 1,129 (54.8%) | 1,137 (55.2%) | 0.8 |
| **Glucose-lowering therapy, n (%)** | 1,625 (27.5%) | 587 (28%) | 1.1 | 563 (27.3%) | 577 (28%) | 1.5 |
| **Antiplatelet therapy, n (%)** | 2,167 (36.7%) | 863 (41.2%) | 9.2 | 825 (40%) | 839 (40.7%) | 1.4 |
| **Anticoagulant, n (%)** | 2,882 (48.8%) | 1,216 (58%) | 18.6 | 1,167 (56.6%) | 1,184 (57.4%) | 1.7 |

**Supplementary Table 10-** Risk of incident AF in the ibrutinib exposed group compared to the acalabrutinib exposed group in matched-patients less or more likely to have a lower or a higher baseline cardiovascular risk of developing AF.

|  | **Ibrutinib** | | **Acalabrutinib** | |  |  |
| --- | --- | --- | --- | --- | --- | --- |
|  | **Number of events** | **Yearly rate, %** | **Number of events** | **Yearly rate, %** | **Risk difference  (95% CI)** | **p value** |
| **Matched-patients with a lower baseline cardiovascular risk of developing AF** | **n=714** | | **n=714** | |  |  |
| **Incident atrial fibrillation** | 31 | 1.2 | 10 | 0.5 | 0.03 (0.01-0.05) | 0.003 |
| **Matched-patients with a higher baseline cardiovascular risk of developing AF** | **n=2061** | | **n=2061** | |  |  |
| **Incident atrial fibrillation** | 386 | 6.1 | 211 | 5.0 | 0.11 (0.08-0.14) | <0.0001 |

p for interaction for the risk of AF in patients less versus more likely to have a lower or a higher baseline cardiovascular risk of developing AF =0.06

**Supplementary Figure 1-** Treatments on Pathway. This figure describes the proportion of patients (before propensity score matching) of the ibrutinib group who switched from ibrutinib to acalabrutinib (panel A) and those of the acalabrutinib group who switched from acalabrutinib to ibrutinib (panel B) during follow-up.


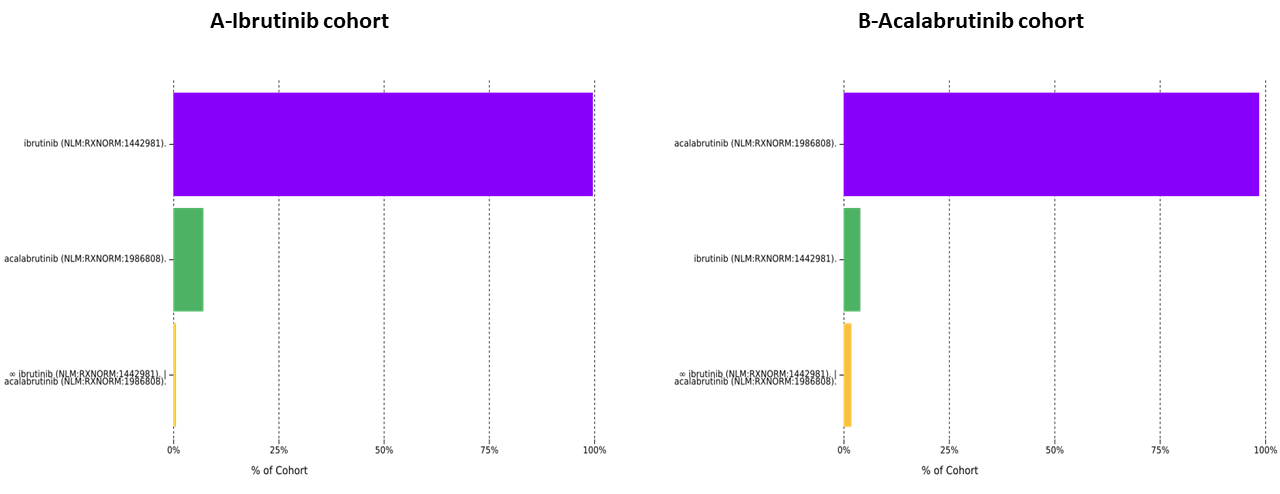


**Supplementary Figure 2-** Time on treatments. This figure demonstrates the time on ibrutinib (panel A) and on acalabrutinib (panel B).


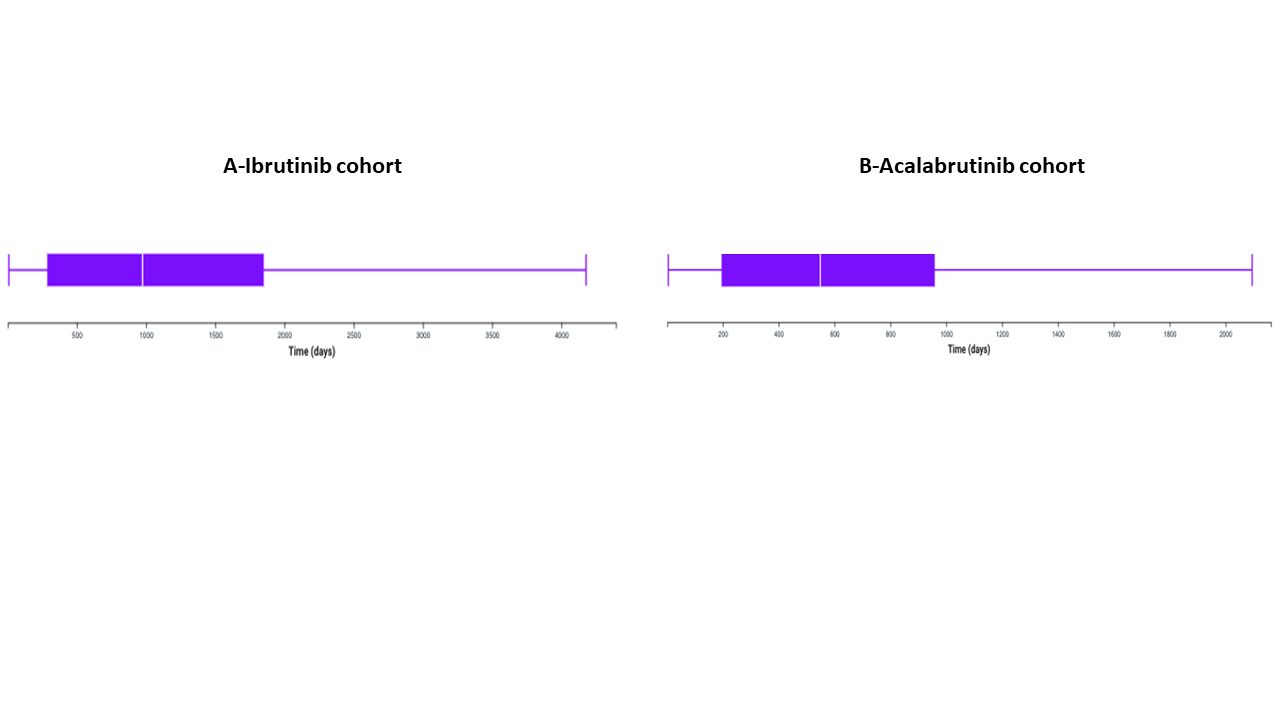


**Supplementary Figure 3-** Propensity Score Matching. This figure demonstrates the cohorts before and after propensity matching (ibrutinib cohort - purple, acalabrutinib cohort - green).


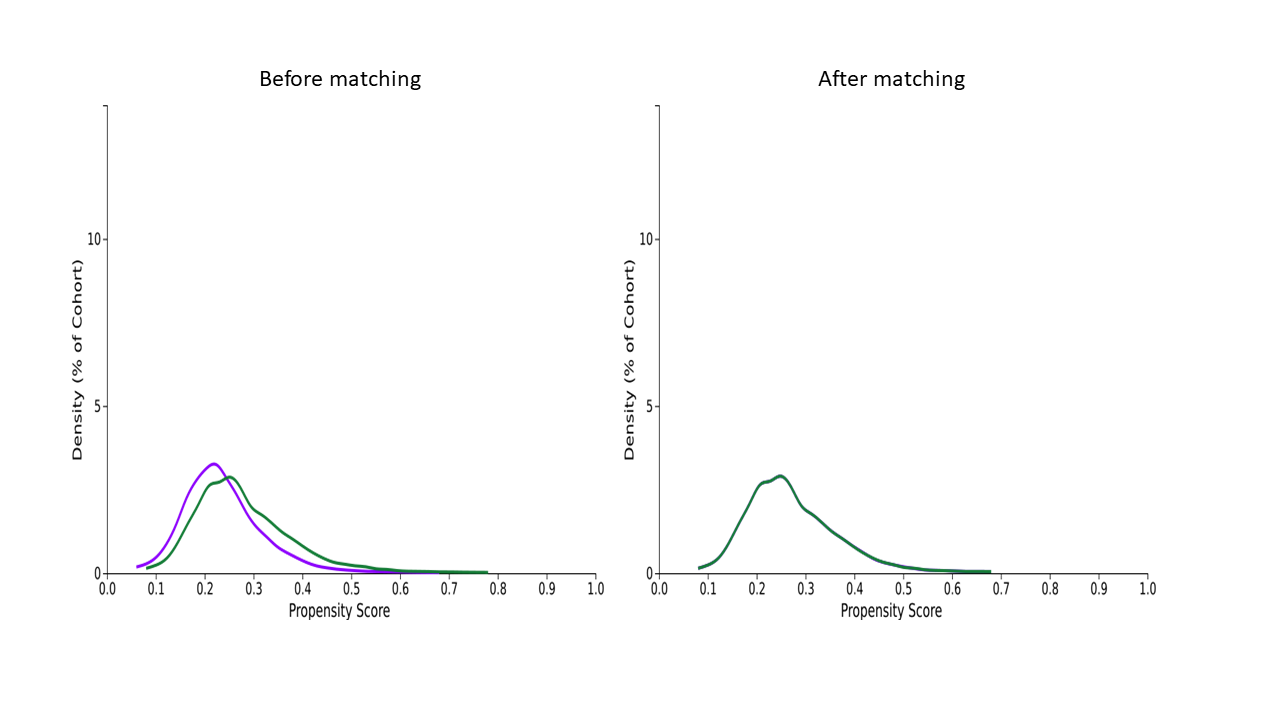

Supplement: Supplementary file 1 — Additional file 1. [file 40164_2025_619_MOESM1_ESM.docx]
